# Prolonged SARS-CoV2 Viral Shedding in an Elderly Patient

**DOI:** 10.7759/cureus.15128

**Published:** 2021-05-19

**Authors:** Roopam Jariwal, Nadia Raza, Michael Valdez, Ayham Aboeed, Ralph Garcia-Pacheco

**Affiliations:** 1 Internal Medicine, University of California Los Angeles David Geffen School of Medicine - Kern Medical Center, Bakersfield, USA; 2 Pulmonary Critical Care, University of California Los Angeles David Geffen School of Medicine - Kern Medical Center, Bakersfield, USA

**Keywords:** covid-19, keywords: case reports, systemic steroids, elderly patients, sars-cov-2 (severe acute respiratory syndrome coronavirus-2)

## Abstract

Coronavirus disease 2019 (COVID-19) has been devastating to the elderly population, especially due to a lack of clear guidelines for treatment. Corticosteroids have been the mainstay in treating the cytokine storm caused by the virus. In the past, prolonged viral shedding of Middle East Respiratory Syndrome (MERS) was noted in patients treated with high-dose corticosteroids. It is unclear whether this also holds true for severe acute respiratory syndrome coronavirus (SARS-CoV2). To our knowledge, this case report highlights the longest reported disease course of SARS-CoV2, lasting approximately 210 days.

## Introduction

The severe acute respiratory syndrome coronavirus 2 (SARS-Cov2) is a novel coronavirus that causes coronavirus disease 2019 (COVID-19). It was first identified in Wuhan, China, in 2019, and since this time, it has spread throughout the world. The transmission risk is still under investigation; however, the person-to-person spread of the virus has been found to occur in the form of droplet transmission. The incubation period of COVID-19 is thought to be within 14 days after exposure with most cases occurring four to five days [1]. Although there are no specific features that reliably differentiate COVID-19 from other viral respiratory illnesses, most patients who are positive for the virus have fever, cough, and fatigue [1]. Guidelines for isolation precautions have evolved since the beginning of the pandemic; however, the duration of these precautions remains controversial in patients with prolonged positivity of viral reverse-transcriptase polymerase chain reaction (RT-PCR).

## Case presentation

**Figure 1:**
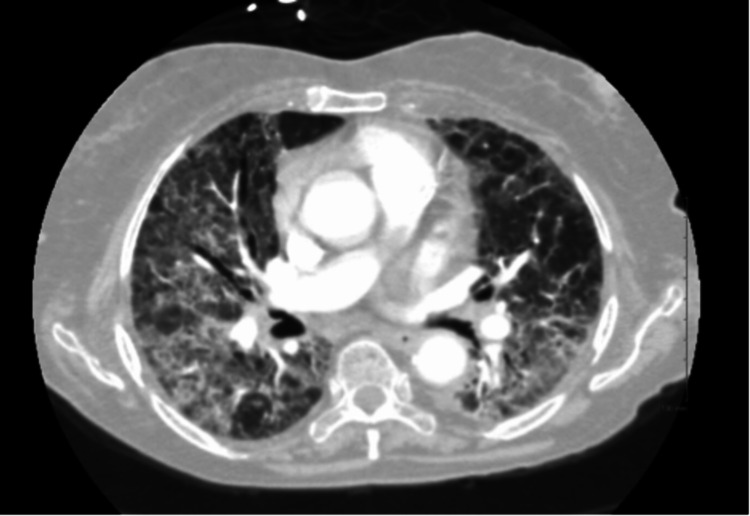
Computed tomography (CT) chest with contrast from initial admission

A 78-year-old Arabic female with a history of lymphoma in 2015 presented to our institution in July 2020 with dyspnea and cough. Prior to this presentation, nasopharyngeal swab polymerase chain reaction (PCR) for SARS-CoV-2 was positive in April 2020, at which time the patient was admitted to a different hospital. During this initial hospitalization, the patient's temperature was 37.3 °C, heart rate was 94 beats/min, respiratory rate was 23 breaths/min, blood pressure (BP) was 146/74 mmHg, and oxygen saturation was 83% on room air, which improved to 94% with supplemental oxygen. Lung examination was notable for bilateral coarse breath sounds. The patient was hypoxic and required supplemental oxygen. The white blood cell (WBC) count was 3,100/mL, with an absolute lymphocyte count of 400/mL, hemoglobin level was 10.2 g/dL, and platelet count was 250,000/mL, with a total protein of 6.2 g/dL. Nasopharyngeal PCR testing for COVID-19 was positive. Computed tomography (CT) chest with contrast (Figure 1) demonstrated diffuse peripheral interstitial and alveolar airspace opacities most prominent at the lung bases and testing for SARS-CoV-2 was again positive. She received supportive care, dexamethasone with prednisone taper upon discharge. After her discharge, her condition improved temporarily; however, she continued to require multiple hospital admissions at different facilities between August and October for similar complaints. During one of the hospitalizations, she was found to be severely immunocompromised, with critically low immunoglobulin G (IgG) levels with a cluster of differentiation 4 (CD4) lymphocyte count of less than 50, human immunodeficiency virus (HIV) antigen/antibody screen negative. Though she received a course of intravenous immunoglobulin (IVIG) afterward, she experienced a gradual decline in her functional capacity between July and October due to her illness. Late in October, she was admitted again to our institution; however, now in the intensive care unit (ICU) requiring mechanical ventilation for hypoxemic respiratory failure. The WBC count was 16,800/mL with an absolute lymphocyte count of 100/mL, hemoglobin level was 9.6 g/dL, and platelet count was 206,000/mL, and total protein of 5.3 g/dL. Nasopharyngeal polymerase chain reaction (PCR) testing for COVID-19 was positive again. 

**Figure 2:**
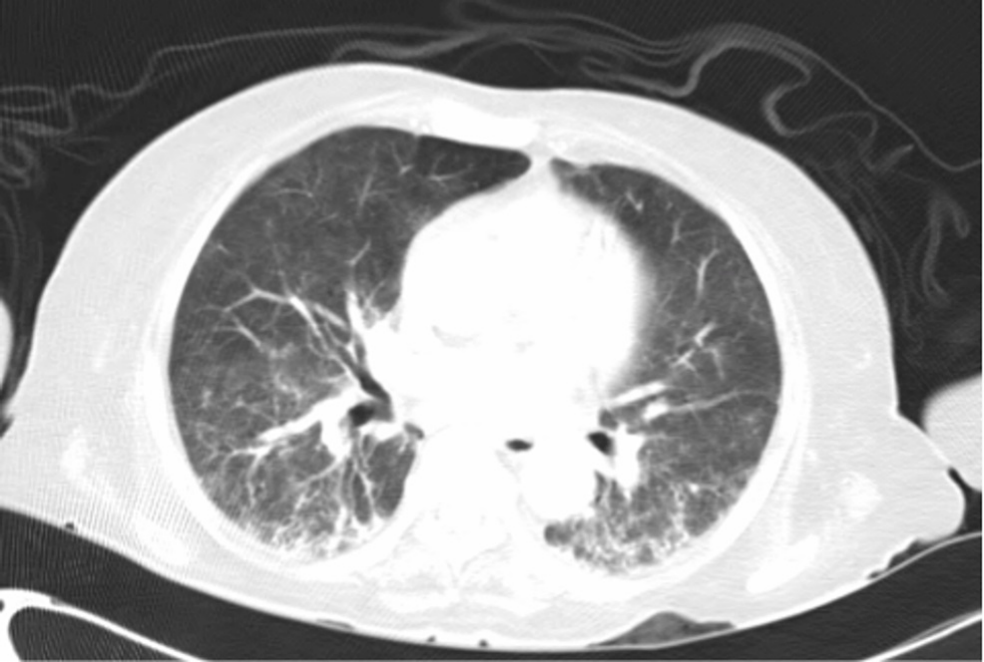
Repeat computed tomography of the chest

Repeat CT scan of the chest (Figure 2) showed significant improvement but residual consolidations with surrounding ground-glass opacities predominantly involving bilateral lung bases. Testing for SARS-CoV-2 was again positive on this admission. Bronchoscopy with bronchoalveolar lavage was performed that revealed inoculation of aspergillus species. Her condition continued to deteriorate, and she subsequently expired. Her clinical course lasted approximately seven months, requiring multiple admissions to different hospitals with multiple courses of corticosteroid therapy in the form of dexamethasone, hydrocortisone, and prednisone.

## Discussion

COVID-19 has been devastating to the elderly population, especially due to the lack of clear guidelines for treatment. Corticosteroids have been the mainstay in treating the cytokine storm caused by the virus. In the past, prolonged viral shedding of Middle East Respiratory Syndrome (MERS) was noted in patients treated with high-dose corticosteroids. It is unclear whether this also holds true for SARS-CoV2. To our knowledge, this case report highlights the longest reported disease course of SARS-CoV2 - lasting approximately 210 days.

The risk of transmitting SARS-CoV-2 begins before an individual develops symptoms and is hypothesized to be the highest in the initial seven to 10 days of the course of the illness, when viral RNA levels are most present in the upper respiratory tract [1]. Prolonged viral shedding, in terms of RT-PCR positivity, was reported in some case reports as ranging from 60-73 days [2-4]. A retrospective study by Xu K et al. found that male gender, delayed hospital admission, and mechanical ventilation were the strongest risk factors contributing to prolonged SARS-CoV2 viral shedding [5]. Our patient did not fall into either category and yet had the longest course of RT-PCR positivity reported in the literature. The same study also looked into the role of corticosteroids; however, they did not consider their role to be significant, as all severe cases of SARS-CoV2 pneumonia were treated with steroids [5]. Soon after, a rebuttal to this study was published that showed that the role of steroids in viral shedding may be dose-dependent and, in fact, high-dose steroids were found to be associated with delayed viral shedding [6]. Our patient received multiple courses of corticosteroids, possibly leading to her prolonged positivity. This makes it more prudent that a randomized controlled trial is needed to investigate whether it is the dose or the duration of corticosteroid administration that plays a role in prolonged viral shedding. 

SARS-CoV2 is known to cause lymphopenia and low CD4+ cell counts [7]. A robust immune system maintains a stable balance between naive and mature CD4+ cells. However, in severe cases of COVID-19, a significant imbalance has been noted in the CD4+ naive to memory cells ratio [7]. This exaggerated response in severe cases is due to the consumption of CD4+ and CD8+ cells and the overproduction of proinflammatory cytokines that dysregulates our innate immunity towards the virus [7]. This patient, a day prior to her demise, was found to have a CD4+ count of 34 cells/mcL, and a CD8+ count of 88 cells/mcL. She was clearly immunocompromised; however, based on the imaging findings (Figure 2), the aspergillus species found on her bronchoalveolar lavage was likely due to inoculation and not an invasive disease. We hypothesize that her immunosuppression was likely due to the prolonged disease course from SARS-CoV2, as she was already under remission from lymphoma and tested negative against other viruses such as HIV.

In another study performed in Wuhan, China, 120 hospitalized COVID-19 patients were studied for risk factors for prolonged viral shedding. Sixty-five percent of these patients received lopinavir/ritonavir (LPV/R), and it was concluded that older age and the lack of treatment with LPV/R were risk factors for prolonged shedding. Older age is the single most independent risk factor for prolonged positivity of SARS-CoV2 RT-PCR, even correlating with progression to acute respiratory distress syndrome and an increase in mortality. Possibly, the lack of robust innate immunity also predisposes elderly patients to prolonged viral shedding [8].

It remains unclear whether age, immunosuppression, the use of corticosteroids, or a combination of all causes prolonged shedding of SARS-CoV2. Further research is needed to understand the immune response caused by SARS-CoV2, especially in the geriatrics population. The Centers for Disease Control and Prevention no longer recommends continuing isolation past 10 days based on RT-PCR results; however, a test-based strategy is recommended for severely immunocompromised patients with persistence of positive viral diagnostic testing [9].

## Conclusions

COVID-19 may cause a dysregulated immune response in elderly patients; this along with an extended course of corticosteroids may contribute to a prolonged disease course. A clearer understanding of the role of longer courses of steroids is imperative to set treatment guidelines, especially in frail, elderly patients. Establishing clear isolation guidelines for patients with prolonged positivity of nasopharyngeal RT-PCR for SARS-CoV2 is crucial to decrease the psychosocial burden of isolation in the patient and family members and exhaustion in healthcare workers.
